# Identification of novel oncogenes in oral cancer among elderly nonsmokers

**DOI:** 10.1002/cre2.739

**Published:** 2023-06-05

**Authors:** Hitoshi Inoue, Masataka Hirasaki, Yasunao Kogashiwa, Yutaka Nakachi, Kiyomi Kuba, Yasuhiro Ebihara, Mitsuhiko Nakahira, Masanori Yasuda, Akihiko Okuda, Masashi Sugasawa

**Affiliations:** ^1^ Department of Head and Neck Surgery, Otolaryngology Saitama Medical University International Medical Center Hidaka Japan; ^2^ Department of Clinical Cancer Genomics Saitama Medical University International Medical Center Hidaka Japan; ^3^ Department of Molecular Brain Science, Graduate School of Medical Sciences Kumamoto University Kumamoto Japan; ^4^ Department of Diagnostic Pathology Saitama Medical University International Medical Center Hidaka Japan; ^5^ Division of Biomedical Sciences, Research Center for Genomic Medicine Saitama Medical University Hidaka Japan

**Keywords:** elderly, *HECTD4*, nonsmokers, oral cancer

## Abstract

**Objectives:**

In recent years, an increase in oral cancer among elderly nonsmokers has been noted. The aim of this study was to identify novel oncogenes in oral cancer in older nonsmokers.

**Material and Methods:**

Whole‐exome sequencing (WES) data from 324 oral cancer patients were obtained from The Cancer Genome Atlas. Single nucleotide variants (SNVs) and insertions/deletions (INDELs) were extracted from the WES data of older patients. Fisher's exact test was performed to determine the specificity of variants in these genes. Finally, SNVs and INDELs were identified by target enrichment sequencing.

**Results:**

Gene ontology analysis of 112 genes with significant SNVs or INDELs in nonsmokers revealed that nonsynonymous SNVs in *HECTD4* were significantly more frequent in nonsmokers than in smokers by target enrichment sequencing (*p* = .02).

**Conclusions:**

Further investigation of the function of *HECTD4* variants as oncogenes in older nonsmokers is warranted.

## 1 INTRODUCTION

Cancer of the oral cavity is one of the most common malignancies (Jemal et al., 2011). Head and neck cancer, including oral squamous cell carcinoma (OSCC), is the sixth most common cancer worldwide, with an estimated 300,400 cases and 145,400 OSCC‐related deaths occurring in 2012 (Torre et al., 2015). Squamous cell carcinoma (SCC) is the most common histology and the main etiological factors are tobacco and alcohol use (Blot et al., 1988).

Smoking is the most important risk factor for oral cancer, and most oral cancers have been considered to arise in smokers. However, epidemiological studies have recently identified an increasing incidence of oral cancer in nonsmokers (Dahlstrom et al., 2008; Farshadpour et al., 2007). By age group, the incidence of oral cancer is increasing in the 50‐ to 79‐year‐old stratum (Ellington et al., 2020). In addition, oral cancer among nonsmoking women has been increasing from the youngest to the oldest age groups (Satgunaseelan et al., 2020). The number of elderly nonsmokers with oral cancer is thus expected to increase in the future.

The causes for this increasing incidence of oral cancer among elderly nonsmokers remain unclear. We hypothesized that this epidemiologically distinct disease would also prove to be genomically distinct, particularly with respect to alterations caused by smoking, and that a better understanding of the differences would identify novel opportunities for treatment and/or primary prevention.

To clarify oncogenes in elderly nonsmokers, the results of whole‐exome sequencing (WES) of The Cancer Genome Atlas (TCGA) were examined for the specificity of single‐nucleotide variants (SNVs) in two groups of oral cancer patients: elderly nonsmokers and elderly smokers. Fisher's exact test showed the specificity of SNVs for 112 genes in the nonsmoker group, so we selected 21 genes for target enrichment sequencing in our elderly clinical cases, as reported here.

## 2 MATERIALS AND METHODS

### 2.1 Retrieval of public data

**Figure 1:**
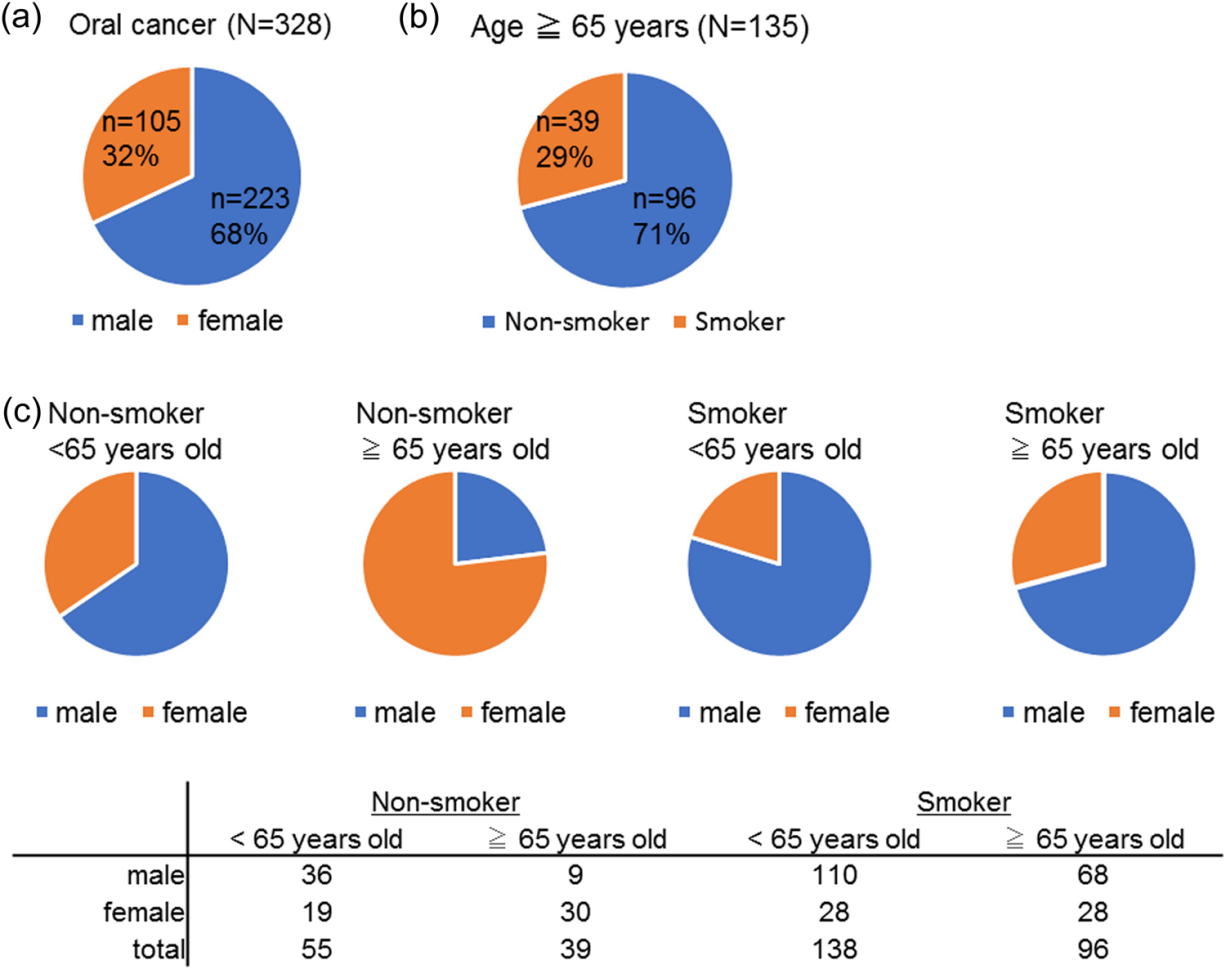
As a result of the analysis of the TCGA data, those aged 65 years and older were defined as elderly, and classified into smoking and nonsmoking groups by gender. (a) The number of male and female components of the 328 anonymized primary oral cancer patients identified in the TCGA database. (b) The smoking trends of primary oral cancer patients aged 65 years and older identified in the TCGA database were shown. (c) Four groups were created according to age and smoking propensity, and the gender ratio of each group was shown. TCGA, The Cancer Genome Atlas.

A total of 328 anonymized patients bearing primary oral cancers were identified from the TCGA database. Clinical patient files were downloaded from the TCGA database website (Firebrowse, http://firebrowse.org/). Of these 328 oral cancer patients, individuals ≥65 years old were defined as elderly and classified by sex and into smoker and nonsmoker groups (Figure 1).

### 2.2 Statistical analysis

Fisher's exact test was performed to determine the specificity of gene SNVs between smokers and nonsmokers (R package; https://bioconductor.org/packages/release/-bioc/html/edgeR.html). All statistical tests were two‐sided, with values of *p* < .05 considered significant.

### 2.3 Gene ontology (GO) analysis

GO analyses were conducted using DAVID web tools (https://david.ncifcrf.gov/).

### 2.4 Our clinical oral cancer cases

Our clinical cases represent data from oral cancer cases treated at Saitama Medical University International Medical Center (SMUIMC).

Among oral cancer patients treated at SMUIMC from April 2007 to October 2019, 60 smokers and 60 nonsmokers were randomly selected. Tumor area and DNA content were confirmed by the naked eye and Qbit, and 97 cases were selected after excluding those with extremely low levels of DNA content.

Among the 97 cases selected, 63 cases with stable DNA content were further selected by quality control (QC) and were considered target cases. The clinical characteristics of these 63 cases are shown in Table 1.

**Table 1:** Clinical characteristics of patients.

	Smokers	Nonsmokers	*p* Value
Total	31	32	
Sex			
Male	29	12	.000356
Female	2	20	
Age (years)			
Mean	72.5	75.5	.0459
Clinical stage			
I, II	11	15	.822
III, IV	20	17	
Primary site			
Tongue	30	28	.329
Floor of mouth	1	4	

The study was approved by the institutional review board at SMUIMC (approval no. 17‐043).

### 2.5 DNA extraction, quantification, and QC

Tissue sections (thickness, 8 µm) were prepared from paraffin‐embedded specimens generated before surgery or from diagnostic biopsy specimens obtained before starting clinical therapy. One of two successive sections was stained with hematoxylin and eosin (HE) to assess the cancerous portion. Manual microdissection using a scalpel and microscope was conducted to recover cancerous portions using the HE‐stained slide as a reference.

Chromosomal DNA was isolated from formalin‐fixed paraffin‐embedded (FFPE) tissues of patients (*n* = 63) with head and neck squamous cell carcinoma (HNSCC) following the instructions from the kit manufacturer (Maxwell RSC DNA FFPE kit; Promega). The concentration of extracted DNA was determined by the fluorometer of the Qubit dsDNA kit (Thermo Fisher Scientific).

The integrity score (ΔΔ*C*_q_ values) and concentration of extracted chromosomal DNA were measured using an Agilent NGS FFPE QC kit (Agilent Technologies) for all samples. As described in the Agilent protocol for HaloPlex HS Target Enrichment, the amount of input DNA was determined based on ΔΔ*C*_q_ values. For ΔΔ*C*_q_ < 1.5, 50 ng was used, and for ΔΔ*C*_q_ > 1.5, 100 ng was used.

### 2.6 Targeted capture and sequencing

A library of the entire genomic sequence of all 21 known genes (*ARHGDIA, BAG3, CACNA2D1, CACNA2D4, CACNG4, CDH18, CUBN, DCHS2, DGKK, EPG5, FAM155A, GRIN3B, HDAC4, HECTD4, IL1RAPL1, MAP3K1, NFKB1, PCDH19, PCDH9, PCDHA1*, and *TRPM3*) was prepared using HaloPlex HS Target Enrichment kits (Agilent Technologies), according to the instructions from the manufacturer. The amount of DNA determined by the criteria described above was fragmented using restriction enzymes. Probes with sequence indexes were added and hybridized to the targeted fragments. Each probe was an oligonucleotide designed to hybridize to both ends of a targeted DNA restriction fragment, thereby guiding the targeted fragments to form circular DNA molecules. The HaloPlex probes were biotinylated, and the targeted fragments were then retrieved with magnetic streptavidin beads. Small fragments and unligated probes were removed from the mix by AMPure purification (Agencourt Bioscience). Next, the circular molecules were closed by ligation. Finally, enriched DNA fragments were amplified using universal primers. The concentration of the enriched library was estimated using a library quantification kit (Kapa Biosystems). High‐throughput sequencing was performed with 100‐bp paired‐end reads on a MiSeq platform (Illumina) for each enriched library according to the protocols from the manufacturer.

### 2.7 Sequence data analysis

The raw sequence read data passed the quality checks in FastQC (http://www.bioinformatics.babraham.ac.uk/projects/fastqc) Read trimming via base quality was performed using FASTX‐toolkit v.0.0.14 (Bolger et al., 2014).

Read alignments to the GRCh38/hg38 were performed using the Burrows‐Wheeler Aligner (Li & Durbin, 2009). Nonmappable reads were removed using SAMtools (Li et al., 2009) After filtering out those reads, we applied the Genome Analysis Toolkit (GATK) (McKenna et al., 2010) local realignment, base quality score recalibration, and SNV and small INDEL discovery. Coverage of the targeted regions was estimated using the GATK DepthOfCoverage. In this experiment, we used SelectVariants to choose variants with “DP > 10” (depth of coverage greater than 10×).

Detected variants were annotated using ANNOVAR software (Wang et al., 2010). Nonsynonymous SNVs identified by targeted sequencing were evaluated using Sorting Intolerant From Tolerant (SIFT) and Polymorphism Phenotyping (PolyPhen2) computational software. Filtering parameters were SIFT version 2 score (ljb2_sift) < 0.05 and PolyPhen version 2 score (ljb2_pp2hdiv) > 0.957.

## 3 RESULTS

### 3.1 TCGA data analysis

A total of 328 anonymized patients bearing primary oral cancers identified from the TCGA database included WES data (Figure 1a). We defined elderly individuals as ≥65 years old and focused on 135 cases from the 328 cases extracted from the TCGA (Figure 1b). TCGA data for the 135 cases of elderly oral cancer showed frequencies of 29% for smokers (*n* = 39) and 71% for nonsmokers (*n* = 96). Proportions of sex differences in smoking prevalence and age were examined. The results showed that the proportion of females was higher in the elderly nonsmoker group than in other groups (Figure 1c).

### 3.2 Extraction of particular SNVs

**Figure 2:**
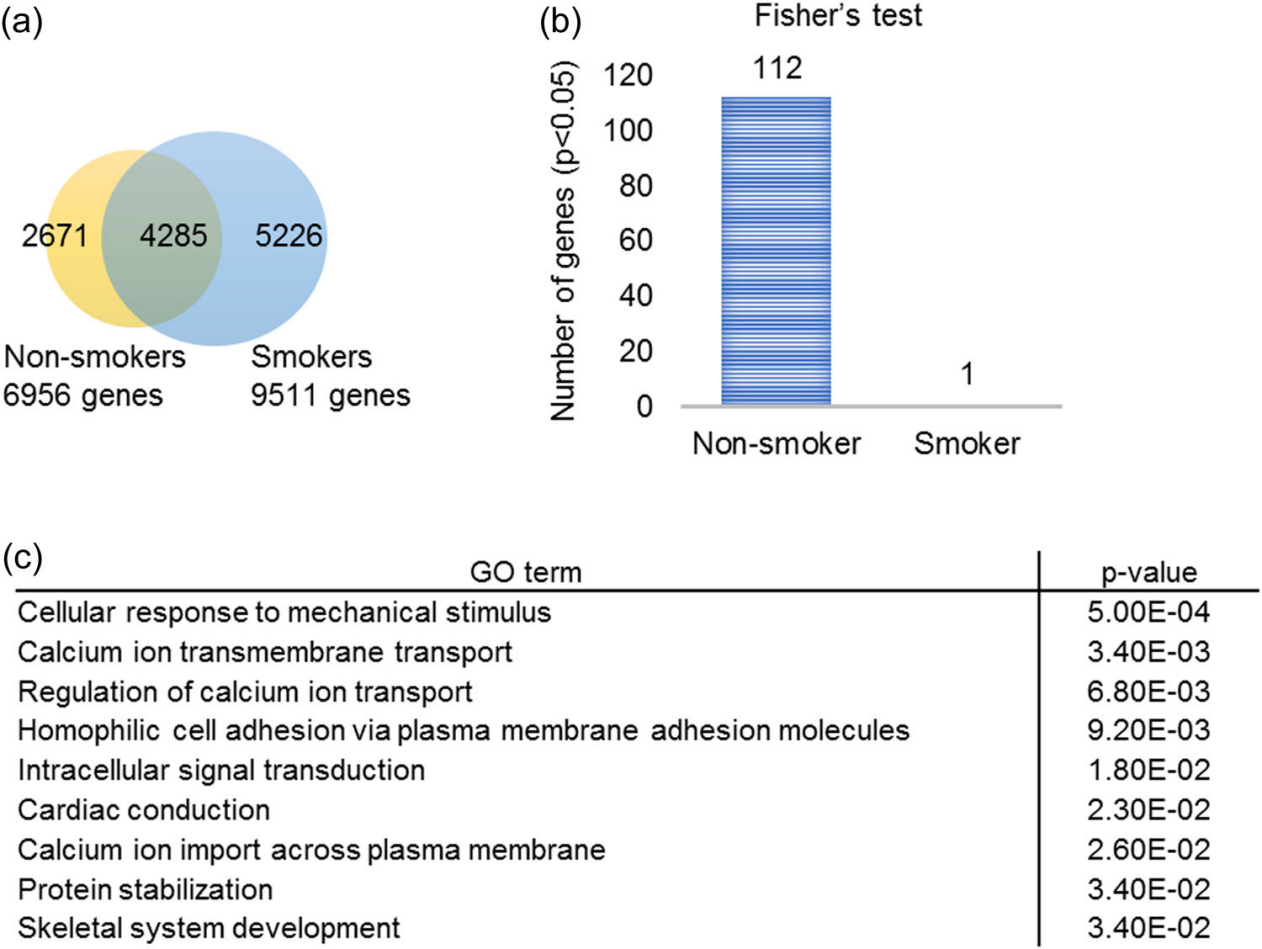
Results of GO analysis of 112 genes with significant SNVs in the nonsmoker group. (a) SNVs or INDELs were detected in 6956 genes in nonsmoker and 9511 genes in smokers. The commonality of each gene was represented by a Venn diagram. (b) Genes with SNVs or INDELs were tested for specificity in smoking tendency using Fisher's exact test. (c) The results of GO analysis of the genes for which SNVs or INDELs were found specifically in nonsmokers are shown. GO, gene ontology; INDEL, insertion/deletion; SNV, single nucleotide variant.

To identify novel oncogenes for oral cancer in elderly nonsmokers, we extracted genes that exhibited SNVs and INDELs from the WES data of TCGA in elderly oral cancer patients. SNVs and INDELs were found in 9511 genes in smokers and 6956 genes in nonsmokers (Figure 2a). Fisher's exact test was performed to determine the specificity of SNVs and INDELs of genes between smokers and nonsmokers. SNVs and INDELs of 112 genes were found to be specific to the nonsmoker group, while SNVs of one gene were found in the smoker group (Figure 2b).

### 3.3 Functional inference by GO analysis

GO analyses were performed to predict the function of the 112 genes that showed significant SNVs and INDELs in the nonsmoker group (Figure 2b). The results suggested that these genes may be involved in “cellular response to mechanical stimulus” (*p* = .0005) and “calcium ion transmembrane transport” (*p* = .0034) (Figure 2c). Abnormality of these functions may cause carcinogenesis of oral cancer in elderly nonsmokers.

### 3.4 Target sequence in our clinical oral cancer cases

We conducted analyses to identify SNVs and INDELs by original target enrichment sequencing in our elderly clinical cases. The clinical characteristics of the 63 cases in our hospital are shown in Table 1. Next, 21 genes were selected (Table 2), including 17 genes in which SNVs were significant in the TCGA results and for which functions were presumed by GO analysis: five genes (*BAG3, ARHGDIA, HDAC4, MAP3K1*, and *NFKB1*) that contribute to “cellular response to mechanical stimulus,” seven genes (*IL1RAPL1, TRPM3, CACNG4, GRIN3B, CACNA2D1, CACNA2D4*, and *FAM155A*) that contribute to “calcium ion transmembrane transport,” five genes (*DCH18, DCHS2, PCDH19, PCDH9*, and *PCDHA1*) that contribute to “homophilic cell adhesion via plasma membrane adhesion molecules,” three genes (*DGKK, EPG5*, and *HECTD4*) with the lowest *p* values from Fisher's exact test and *CUBN* as the only gene that showed a significant difference in smokers (Figure 2b; Table 2).

**Table 2:** Results of genes that were significantly variant in the nonsmoking group for each GO term.

	Nonsmoker Alt	Nonsmoker Ref	Smoker Alt	Smoker Ref	*p* Value
*Cellular response to mechanical stimulus*					
*BAG3*	3	36	0	95	.02331
*ARHGDIA*	3	36	0	95	.02331
*HDAC4*	3	36	0	95	.02331
*MAP3K1*	3	36	0	95	.02331
*NFKB1*	3	36	0	95	.02331
*Calcium ion transmembrane transport*					
*IL1RAPL1*	4	35	0	95	.00641
*TRPM3*	6	33	2	93	.00772
*CACNG4*	3	36	0	95	.02331
*GRIN3B*	3	36	0	95	.02331
*CACNA2D4*	3	36	0	95	.02331
*FAM155A*	4	35	1	94	.02513
*CACNA2D1*	5	34	3	92	.04597
*Homophilic cell adhesion via plasma membrane adhesion molecules*					
*CDH18*	4	35	1	94	.02513
*DCHS2*	4	35	1	94	.02513
*PCDH19*	3	36	0	95	.02331
*PCDH9*	4	35	1	94	.02513
*PCDHA1*	5	34	2	93	.02206
*Lowest p value genes in Fisher's exact test and the only gene that showed a significant difference in smokers*					
*EPG5*	5	34	0	95	.00172
*HECTD4*	7	32	2	93	.00256
*DGKK*	7	32	2	93	.00256
*CUBN*	0	39	16	79	.00326

Abbreviations: Alt, any other allele found at that locus; GO, gene ontology; Ref, allele in the reference genome.

Target regions were designed to enrich exonic regions including the 5′‐untranslated region (UTR) and 3′‐UTR and exon–intron junctions of all 21 genes (Supporting Information: Table 1). The mean percentile of covered target regions was 97.72%.

### 3.5 Quality assessment

**Figure 3:**
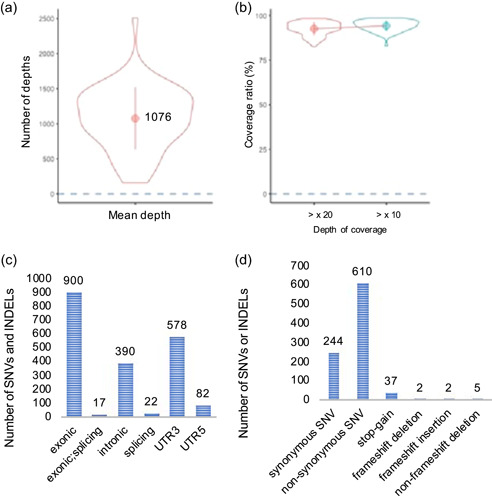
Result of original target enrichment sequencing in our elderly clinical cases. (a) The trend of the mean depth for each of the 63 multiplexed samples is shown in the violin plot. (b) The trend of the coverage ratio for each of the 63 multiplexed samples is shown in the violin plot. (c, d) The number of SNVs or INDELs identified by original target enrichment sequencing is shown. Classification was performed for each of the six regions (c). Classification was performed by variant type (d). INDELs, insertions/deletions; SNV, single nucleotide variant.

A median of 3,493,323 sequence‐mapped reads was obtained per sample (range, 437,058–6,034,492 reads/sample). Of the designed target bases, 92.5% (range, 82.6–98.3%/sample) had at least 20‐fold coverage, with a mean coverage of 1077‐fold (range, 159‐ to 2509‐fold) per nucleotide in the coding region of the target gene (Figure 3a,b).

### 3.6 Evaluation of nonsynonymous SNVs

As a result of original target enrichment sequencing in our elderly clinical cases, 1989 SNVs or INDELs were found in target regions (Figure 3c). There were 610 nonsynonymous SNVs in amino acid sequences, 2 with frameshift deletions and 2 with insertions (Figure 3d). SNVs showing the stop‐gain variant showed 37 locations (Figure 3d). The results of Fisher's exact test between smoker and nonsmoker groups showed that SNV (*p* = .0424) in the 3′‐UTR of *BAG3* was significant in the nonsmoker group (Table 3). On the other hand, the nonsynonymous SNV (*p* = .0413) of *CUBN*, was significantly different among smokers (Figure 2; Table 3).

**Table 3:** Results of targeted enrichment sequencing for individual SNVs.

Gene symbol						Smokers		Nonsmokers		
	SNP IDs	Function	Exonic function	Nucleotide change	aa change	RefSeq (*n*)	AltSeq (*n*)	RefSeq (*n*)	AltSeq (*n*)	*p* Value
*BAG3*	rs3981124	UTR3	NA	NA	NA	26	4	20	12	.0424
*CUBN*	NA	Exonic	Nonsynonymous SNV	NM_001081:c.2695A> T	p.I899L	20	11	28	4	.0413
*DCHS2*	rs61743677	Exonic	Nonsynonymous SNV	NM_001358235:c.8789A > G	p.K2930R	23	8	30	2	.0433
*DCHS2*	rs61746132	Exonic	Nonsynonymous SNV	NM_001358235:c.9392C > T	p.P3131L	23	8	31	1	.0127

*Note*: We performed Fisher's exact test on the SNVs identified in the original target enrichment sequence and only those SNVs that showed superiority are presented.

Abbreviations: aa, amino acid; AltSeq, any other allele found at that locus; RefSeq, the allele in the reference genome; SNV, single nucleotide variant.

In‐silico analyses were only performed for nonsynonymous SNVs that resulted in an amino acid substitution. PolyPhen2 and SIFT were used to predict the effects of missense mutations. The in‐silico prediction programs provide related information which indicates that a particular variant may affect the biological activity of the mature protein. SIFT and PolyPhen2 programs were used to estimate functional effects on the respective encoded proteins. In all cases, both SIFT and PolyPhen2 predicted that the variants would result in functionally impaired protein structures. We also hypothesized that frameshift deletions, frameshift insertions, stop‐gains, and splicing would result in protein structures that were functionally compromised. For each gene with a mutation that was predicted to result in a functionally impaired protein structure as a result of in‐silico analysis, Fisher's exact test was performed between smokers and nonsmokers. The results showed that nonsmokers were significantly more likely to have at least one mutation in *HECTD4* (Table 4). Such results suggest that *HECTD4* may be strongly involved with carcinogenesis in elderly nonsmokers.

**Table 4:** Results of targeted enrichment sequencing for individual genes.

	Smokers		Nonsmokers		
Gene symbol	RefSeq (*n*)	AltSeq (*n*)	RefSeq (*n*)	AltSeq (*n*)	*p* Value
*HECTD4*	7	24	1	31	.0265
*CACNA2D4*	26	5	31	1	.1042
*PCDH9*	26	5	31	1	.1042
*PCDH19*	25	6	30	2	.1477
*GRIN3B*	2	29	6	26	.2565

*Note*: Fisher's exact test was performed for each gene harboring SNVs that were predicted to be damaged by SIFT and PolyPhen2. The five genes with the lowest *p* values are shown. RefSeq means the allele in the reference genome. AltSeq means any other allele found at that locus.

Abbreviations: aa, amino acid; AltSeq, any other allele found at that locus; RefSeq, the allele in the reference genome; SIFT, Sorting Intolerant From Tolerant; SNV, single nucleotide variant.

### 3.7 Analysis of clinical data

No significant differences were seen in survival or treatment response according to the presence or absence of *HECTD4* variants.

Regarding the effect of alcohol consumption, *HECTD4* variants were slightly more common in the group without a history of alcohol consumption, but the difference was not significant. However, this suggested that *HECTD4* may not be affected by environmental factors.

Within the range of cases available for investigation, no associations between the presence or absence of the human papilloma virus (HPV) or the frequency of *HECTD4* variants were found. However, the small number of cases made it difficult to draw conclusions.

### 3.8 Deletion of short tandem repeat (STR) regions in elderly patients with oral cancer

STR sequences (including microsatellite sequences) are repetitive sequences found in the genome of the cell nucleus or organelles that consist of a repetition of a unit sequence of a few bases. High‐frequency microsatellite instability (MSI‐High) is a condition in which the number of microsatellite repeats is abnormally high. Although microsatellite abnormalities do not necessarily lead to cancer, tissues that show MSI‐high are considered cancer‐prone (Nojadeh et al., 2018). Frameshift deletion of *DCHS2* (rs140019361), an STR site that repeats TTTG six times, was found in 95% of oral cancers in elderly individuals, both smokers and nonsmokers (Table 3). Nonframeshift deletion of STR sites in *MAP3K1* (rs570353965) and *FAM155A* (rs3832903) was also found at similarly high rates (Table 5).

**Table 5:** Deletion of Short tandem repeat regions of elder oral cancer.

						Smokers		Nonsmokers		
Gene symbol	SNP IDs	Function	Exonic function	Nucleotide change	aa change	RefSeq (*n*)	AltSeq (*n*)	RefSeq (*n*)	AltSeq (*n*)	*p* Value
*DCHS2*	rs140019361	Exonic	frameshift deletion	NM_001142552:c.4095_4098del	p.N1365Kfs*3	2	29	1	31	.612
*MAP3K1*	rs570353965	Exonic	nonframeshift deletion	NM_005921:c.2828_2830del	p.T949del	12	19	12	19	1.000
*FAM155A*	rs3832903	Exonic	nonframeshift deletion	NM_001080396:c.218_229del	p.Q73_Q76del	15	15	16	16	1.000

Abbreviations: aa, amino acid; AltSeq, any other allele found at that locus; RefSeq, the allele in the reference genome; SNV, single nucleotide variant.

## 4 DISCUSSION

Many reports have described studies on gene mutations in oral cancer. Among these, genetic factors and the mechanism of canceration are being elucidated to some extent. However, their content is due to environmental factors (smoking, alcohol, mechanical irritation) (Ali et al., 2017; Sasahira & Kirita, 2018). On the other hand, studies on young nonsmokers unrelated to environmental factors have shown similar results to those of oral cancer in the elderly (Pickering et al., 2014).

To date, no reports have explained the genetic factors or mechanisms of elderly individuals who are not at risk for developing oral cancer from environmental factors. This study focused on smoking as one of these environmental factors. As the number of such oral cancer cases is expected to increase with the aging of the population, the development of markers for primary prevention and new therapeutic agents is urgently needed. To identify novel oncogenes in oral cancer of oral cancer in elderly nonsmokers, we extracted genes that exhibited SNVs and INDELs from the WES data of TCGA in elderly oral cancer patients. The specificity of SNVs and INDELs of genes between smokers and nonsmokers was examined, and 112 genes were specifically detected in nonsmokers. GO analyses of these 112 genes suggested that they may be involved in “cellular response to mechanical stimuli” and “transmembrane transport of calcium ions.”

From the analysis of TCGA, we identified 21 genes with significant differences in the number of variant genes between elderly smokers and elderly nonsmokers: *ARHGDIA, BAG3, CACNA2D1, CACNA2D4, CACNG4, CDH18, CUBN, DCHS2, DGKK, EPG5, FAM155A, GRIN3B, HDAC4, HECTD4, IL1RAPL1, MAP3K1, NFKB1, PCDHA1, PCDH19*, *PCDH9*, and *TRPM3*.

Our original targeted sequencing of clinical specimens confirmed the presence of significant mutations in *HECTD4* in elderly nonsmokers. HECTD4 is known for being homologous to the E6‐AP carboxyl terminus (HECT) domain containing E3 ubiquitin protein ligase 4. In prostate cancer, overexpression of HECTD4 has been reported to result in a simultaneous decrease in both androgen receptor (AR) and MYC proteins, while knockdown of HECTD4 results in an increase in both AR and MYC proteins. These results suggest that HECTD4 plays a role as an E3 ligase targeting AR and MYC (Vatapalli et al., 2020). The proto‐oncogene c‐Myc is markedly upregulated in oral cancer patients, and its expression correlates with the clinicopathological grade and stage of oral cancer. In addition, c‐Myc has been reported as an important factor in the development of oral cancer in immunocompromised mice (Wang et al., 2019). AR has also been recognized for its importance in cancer etiology and progression. OSCC cells express AR, and in vitro experiments and patient studies have shown that AR plays an important role in promoting cell growth (Liu et al., 2019). We did not find any reports that described *HECTD4* as a putative driver of OSCC in our searches. However, reports that decreased *HECTD4* in prostate cancer leads to increased AR and MYC proteins and that increased AR/MYC is oncogenic in OSCC suggest that loss‐of‐function mutations in *HECTD4* are oncogenic and may be a cause of carcinogenesis in elderly nonsmokers.

No significant differences were seen in survival or treatment response according to the presence or absence of *HECTD4* variants. Due to the small number of cases in this study, the correlation between *HECTD4* mutations and prognosis could not be fully tested. However, based on previous reports, *HECTD4* variants are expected to increase MYC and result in a poor prognosis. The presence or absence of *HECTD4* variants may thus play a role in posttreatment selection and whether prophylactic neck dissection should be added in the absence of lymph node metastases. This issue should therefore be examined in more detail in a larger cohort in future research.

To our knowledge, this study is the first to reveal a genetic variant that characterizes elderly nonsmokers with OSCC, particularly in a Japanese cohort. However, some limitations of the present study should be considered. First, the small number of cases in this study may have been the reason that the mutation in *HECTD4* was the only gene mutation to show molecular biological features of oral cancer in older nonsmokers. Second, somatic mutations that accumulate in normal tissues have been linked to aging, disease, and disorders. A recent series of studies reported comprehensive genomic analyses of morphologically normal tissues (Li et al., 2021). Even in morphologically normal tissues, accumulation of somatic mutations and clonal expansion were widely observed to varying degrees. We hypothesized that a comprehensive genomic analysis of normal tissues from smokers and nonsmokers would facilitate an understanding of the carcinogenic risk of oral cancer in elderly nonsmokers. Third, this study was retrospective in design and examined only somatic mutations, so no germline information was examined. In addition, DNA extraction was performed from formalin sections, and the DNA may thus have been degraded or modified to some extent. In the future, we would like to increase the number of cases to investigate whether *HECTD4* has any role as a marker by examining in detail the effects of the presence or absence of *HECTD4* variants on malignancy and prognosis in clinical specimens.

## 5 CONCLUSION

We identified a significant variant in *HECTD4* in elderly nonsmokers, compared with elderly smokers. Further study is warranted to elucidate whether *HECTD4* variants may lead to the identification of therapeutic targets.

## AUTHOR CONTRIBUTIONS

**Hitoshi Inoue**: Writing—original draft preparation; data curation. Yasunao Kogashiwa: Conceptualisation, formal analysis, Writing—original draft preparation, funding acquisition. **Masataka Hirasaki**: Analysis, Writing—original draft preparation. **Yutaka Nakamichi**: Data analysis and interpretation. **Kiyomi Kuba**: Data acquisition. **Yasuhiro Ebihara**: Statistical analysis. **Masanori Yasuda**: Data acquisition. Mitsuhiko Nakahira, **Akihiko Okuda and Masashi Sugasawa**: Supervision. All authors reviewed the manuscript.

## CONFLICT OF INTEREST STATEMENT

The authors declare no conflict of interest.

## ETHICS STATEMENT

This study was approved by the institutional review board at SMUIMC (Title: Study to elucidate carcinogenic factors of oral cancer in elderly and nonsmokers; approval no. 18‐280; date of approval: February 13, 2019). Due to the retrospective nature of this study, the requirement for informed consent was waived by the institutional review board at SMUIMC.

## Supporting information

Supporting information.

Supporting information.

## Data Availability

The data that support the findings of this study are available from the corresponding author upon reasonable request.
