# Supplementary material for: Identification of novel oncogenes in oral cancer among elderly nonsmokers
Source: Clin Exp Dent Res. 2023 Jun 5;9(4):711–20. doi: 10.1002/cre2.739 (PMC10441604; doi:10.1002/cre2.739)
Supplement: Supplementary file 1 — Supporting information. [file CRE2-9-711-s001.pdf]

FORTE

Forte Science Communications

Forte, Inc., KDX Shinjuku 286 Bldg. 5F, Shinjuku 2-8-6, Shinjuku-ku, Tokyo 160-0022, Japan
Tel: +81-3-3353-3545 Fax: +81-3-3354-3845 Email: info@forte-science.co.jp

March 3, 2023

To Whom It May Concern:

This is to confirm that Dr. Kogashiwa’s manuscript entitled "Identification of novel oncogenes in oral cancer among elderly non-smokers" has been edited by a native English speaker at Forte, Inc. Forte is a Japan-based company that employs editors and rewriters with a science background. We have been helping the Japanese scientific community to publish in international journals since 1987.

While this certificate confirms the authors have used Forte’s editing services, we cannot guarantee that additional changes have not been made after our edits. Should you require any additional information about our services, please do not hesitate to contact our office.

Sincerely,

Forte, Inc.

株式会社フォルテ之印

KDX Shinjuku 286 Bldg. 5F
2-8-6 Shinjuku, Shinjuku-ku
Tokyo 160-0022, Japan
Tel: +81-3-3353-3545
Fax: +81-3-3354-3845
Email: info@forte-science.co.jp
