# Supplementary material for: Identification of novel oncogenes in oral cancer among elderly nonsmokers
Source: Clin Exp Dent Res. 2023 Jun 5;9(4):711–20. doi: 10.1002/cre2.739 (PMC10441604; doi:10.1002/cre2.739)
Supplement: Supplementary file 2 — Supporting information. [file CRE2-9-711-s002.docx]

**Supplementary table 1. Results for target sequences in our clinical oral cancer cases**

| Symbol | Location (hg19) | Target region (bp) | Coverage region (%) |
| --- | --- | --- | --- |
| *ARHGDIA* | chr17:79825585-79829292 | 2,985 | 97.62 |
| *BAG3* | chr10:121410872-121437341 | 2,867 | 96.02 |
| *CACNA2D1* | chr7:81575750-82073132 | 9,878 | 95.91 |
| *CACNA2D4* | chr12:1901113-2028012 | 8,399 | 98.86 |
| *CACNG4* | chr17:64960970-65029528 | 3,510 | 97.83 |
| *CDH18* | chr5:19473050-20575992 | 5,286 | 92.85 |
| *CUBN* | chr10:16865953-17171840 | 14,548 | 98.89 |
| *DCHS2* | chr4:155153389-155412940 | 15,184 | 98.26 |
| *DGKK* | chrX:50108396-50213747 | 7,968 | 99.45 |
| *EPG5* | chr18:43427564-43547315 | 14,640 | 97.7 |
| *FAM155A* | chr13:107820869-108519470 | 3,919 | 98.24 |
| *GRIN3B* | chr19:1000408-1009741 | 3,462 | 99.83 |
| *HDAC4* | chr2:239969854-240323358 | 14,203 | 97.15 |
| *HECTD4* | chr12:112597982-112819906 | 22,765 | 97.39 |
| *IL1RAPL1* | chrX:28605506-29974850 | 3,887 | 97.86 |
| *MAP3K1* | chr5:56110890-56191989 | 8,597 | 98.84 |
| *NFKB1* | chr4:103422476-103538469 | 6,817 | 99.69 |
| *PCDH19* | chrX:99546632-99665281 | 9,876 | 99.85 |
| *PCDH9* | chr13:66876956-67804478 | 7,225 | 95.22 |
| *PCDHA1* | chr5:140165711-140391939 | 5,672 | 97.76 |
| *TRPM3* | chr9:73143969-74061830 | 20,536 | 96.9 |
